# Addressing the COVID-19 Shock: The Potential Job Creation in China by the RCEP

**DOI:** 10.3390/ijerph192315669

**Published:** 2022-11-25

**Authors:** Xinxiong Wu, Chen Chen Yong, Su Teng Lee

**Affiliations:** 1 Institute for Advanced Studies, Universiti Malaya, Kuala Lumpur 50603, Malaysia; 2 Faculty of Business and Economics, Universiti Malaya, Kuala Lumpur 50603, Malaysia

**Keywords:** coronavirus disease (COVID-19), Regional Comprehensive Economic Partnership (RCEP), social accounting matrix (SAM), job creation

## Abstract

In 2020, coronavirus disease (COVID-19) left around 81% of the global workforce, nearly 2.7 billion workers, affected. Employment in China was the first to be hit by COVID-19. The Regional Comprehensive Economic Partnership (RCEP) is expected to bring dynamism to China’s employment market in an era of long COVID-19. This study aims to examine the number of sectoral jobs that the RCEP will create in China, with the number of skilled or unskilled labour employed in each sector. The exogenous shocks to the RCEP can be reflected in the number of jobs created through multipliers based on a social accounting matrix compiled from China’s input-output tables in 2017, combined with the employment satellite accounts compiled. The results show that the RCEP is expected to create over 17 million potential jobs in China, with unskilled labour accounting for 10.44 million and skilled labour for 6.77 million. It is even expected that there will be job losses in the metalworking machinery sector. The contribution of this paper can serve as a reference for policies to protect vulnerable sectors, further open up trade markets and strengthen cooperation among RCEP members as important measures to address the employment impact of long COVID-19.

## 1. Introduction

At the end of 2019, a few cases of unexplained pneumonia were identified in some hospitals in Wuhan, Hubei Province, which were confirmed to be acute respiratory infections caused by the 2019 novel coronavirus infection [1]. On 11 February 2020, World Health Organization Director-General Dr Tedros Adhanom Ghebreyesus announced that the new coronavirus-infected pneumonia would be named COVID-19. COVID-19 has serious implications for the productivity, performance and well-being of the workforce worldwide [2]. COVID-19 led to a sharp decline in labour demand in many sectors of the economy and resulted in significant initial labour shortages in other sectors [3]. COVID-19 left education, training and work-based learning disrupted, with increased difficulties for job seekers and new entrants to the labour market. At the same time job and income losses, as well as the quality of employment, have deteriorated [3,4]. In addition, COVID-19 has a negative psychological impact on the workforce, as people with COVID-19 may be discriminated against in employment and healthy people have a constant fear of contracting COVID-19 at work [5,6]. China’s response to COVID-19 was swift, including blocking the movement of people between cities and quarantining potential patients [7]. COVID-19 has severely impacted the Chinese labour market, making it impossible or difficult for everyone to get to work [8]. By June 2020, there was still 20% of the workforce “lost”, of which 11% were “unemployed”, 4% were “waiting to return to work” and 5% were in employment but with “insufficient workload recovery” [9]. Even in some sectors where remote working is possible, there is still a low level of productivity and well-being [2]. On 24 September 2022, Singapore’s Union-Tribune website reported that Dr Tedros Adhanom Ghebreyesus noted that there is still a long way to go before the COVID-19 outbreak is declared over. Therefore, trade is an important tool in how to face the shock of COVID-19 on employment in China [10,11].

On 1 January 2022, the Regional Comprehensive Economic Partnership (RCEP), a free trade agreement initiated by ASEAN in 2012 consisting of 15 members, including China, Japan, South Korea, Australia, New Zealand, and ten ASEAN countries, entered into force. The RCEP is the world’s largest and most important free trade agreement negotiation, covering nearly one-third of the world’s population and trade volume, making it the world’s most populous free trade area with the largest population, most diverse membership and most dynamic development. However, in the ten years since the Regional Comprehensive Economic Partnership (RCEP) was launched and negotiated, especially in the past few years, the world trade environment has changed dramatically [12,13,14]. International markets, global industrial chains and supply chains have undergone structural changes under the influence of COVID-19. Some of the world’s larger economies have had diverse reactions to the RCEP. The United States has a mixed attitude towards the RCEP, arguing that it strengthens the ties between Asian economies but at the same time intensifies competition between major powers [15,16]. The RCEP is regarded as largely offsetting the significant impact of the US–China trade war on the world at large [12,17]. The negative effects of the RCEP on the European Union (EU) are, on the one hand, that the RCEP will weaken the influence of EU companies and reduce investment in EU companies and, on the other hand, that the RCEP allows the huddling of Asian economies to weaken the influence of EU standards [18,19]. These rapidly changing international environments make China face a complicated environment that it has never encountered before, because China occupies an important position in the global industrial chain and supply chain.

Previous scholars have quantitatively assessed the effects of the RCEP on China at the regional level through quantitative analysis of structural models and the Global Trade Analysis Project model, concluding that labour-intensive sectors are more significantly affected by the RCEP, followed by capital-intensive sectors, and technology-intensive sectors are the weakest [20,21]. Will the RCEP be a perfect agreement for China, a developing country with a labour-intensive workforce of 776.4 million? Not really. For example, in agriculture, China’s employment rate is declining [22,23]. On the one hand, China is currently undergoing a period of rapid industrialisation, with fast-growing industrial capital bringing far greater returns than agriculture, which in turn has led to further expansion of industrial capital. This has led to a steady flow of factors from agriculture to industry, which itself is unable to complete the pricing of its land factor returns and certainly not its labour factor. The fact that agricultural factors are priced by external industry and markets can lead to unprofitable agriculture. Agricultural capital, labour and land flow to cities and industry, and agricultural employment is declining [24]. On the other hand, agricultural output in Australia and New Zealand comes from a large farm model that can generate similar returns to industry, and their agriculture is competitive [25,26,27]. The prices of bulk commodities such as grain and livestock are comparable to international prices in the market. The prices of their products are even lower than the factor costs in China. No labour would be willing to enter agriculture without tariff protection or government subsidies, and Chinese agriculture would be in for a colossal disaster. It is, therefore, crucial to explore the impact of the RCEP as an exogenous shock on the Chinese labour market. The questions that need to be addressed in this research are:

1. How much sectoral job creation is expected from the RCEP in China?
2. Is there more job creation in the skilled or unskilled labour?
3. How is the government seizing the RCEP opportunity to deal with the shock of COVID-19?

## 2. Literature Review

### 2.1. Trade in Goods

The RCEP’s liberalisation of trade in goods is a two-by-two offer of tariff reduction commitments by the 15 member countries. More than 90 percent of intra-regional trade in goods will eventually achieve zero tariffs, including immediate zero tariffs and zero tariffs over the next ten years. This allowsthe RCEP to deliver its promise to liberalise the vast majority of trade in goods in the short term, significantly reducing intra-regional trade costs and commodity prices [28]. For example, Brunei has increased its zero-tariff product commitments to China on tobacco, bedding, home appliances and furniture, Thailand on paper products, copper and electric motors, and Cambodia on chicken, vegetables and fruits, food and rubber. China has also increased its zero-tariff commitments to ASEAN countries on fruits, chemicals, diesel engines, auto parts, etc. According to the Chinese Ministry of Commerce, ASEAN’s actual investment in China’s manufacturing sector reached USD 2.18 billion in 2020, topping all sectors. Next in line are the real estate industry, leasing and business services, transportation, storage and postal services, and wholesale and retail trade, with the number of new enterprises in these four sectors reaching 990 and the actual investment amounting to USD 4.42 billion, accounting for 52.9 percent and 55.5 percent of the total number of new enterprises and actual investment by ASEAN in China, respectively. In terms of commodity structure, electromechanics, plastics and their products, iron and steel and their products, textiles, mineral fuels and transport equipment are the main products traded by China with RCEP members [29]. The RCEP region is the primary import and export market for Chinese mineral fuels, mineral oils and their products, and China’s imports and exports to RCEP members all account for more than 40 percent of the total import and export of this category of goods. The rules of origin accumulation are considered the most significant achievement of the RCEP in the area of trade in goods. Under the cumulative rules of origin, materials of origin from other RCEP member countries can be accumulated to meet the 40 percent value-added origin criterion for final exports when specifying the product’s eligibility for tariff preferences. For example, if a member country supplies the raw materials for a Chinese-made household appliance at more than 40 percent, it will be exempted from 20 percent tariffs when exported to another member country. The RCEP simplifies customs clearance procedures by adopting efficient management tools such as pre-determination, pre-arrival processing, and information technology. Goods are released within 48 h of arrival whenever possible. Express cargo, perishable goods, etc., are released within 6 h whenever possible [30]. This is expected to reduce the overall customs clearance time for goods in the region.

### 2.2. Trade in Services

China’s services trade liberalisation level reaches the highest level of existing free trade agreements [31]. Firstly, the number of committed service sectors has increased by 22 from the approximately 100 sectors committed by China’s accession to the World Trade Organisation, mainly in 11 areas such as management consultancy-related services, manufacturing-related services, professional design services, elderly care services, sports and entertainment services, passenger transport services, market research services, personnel placement services, beauty and hairdressing services, building cleaning services, printing services, and 11 other areas. Secondly, the level of commitment was raised in 37 sectors, mainly in 12 areas such as legal services, construction and engineering services, environmental services, insurance services, banking services, securities services, shipping and related services, real estate services, aircraft maintenance and computer reservation system services, advertising services, software implementation services, and interpretation and translation services. In terms of service sectors that China focuses on, other RCEP member countries have committed to a greater degree of liberalisation in sectors such as construction, engineering, tourism, finance and transport [32]. China has also overall liberalised service sectors related to the installation of computer hardware, wholesale or retail trade services without a fixed location, maritime agency services, and road trucking or motor freight transport services, granting full national treatment to foreign investors. These new areas of liberalisation provide a wide market space for Chinese companies to go global and expand their regional industrial chain [33].

### 2.3. Investment

The investment chapter of the RCEP integrates and upgrades the investment rules of the 10+1 Agreement and provides a comprehensive and balanced investment arrangement in terms of investment market access and investment protection, forming the most extensive investment agreement arrangement in the Asian region and helping to create a more stable, open, and convenient investment environment. All RCEP member countries have adopted the negative list model to make commitments in terms of investment market access [34]. China has made high-level liberalisation commitments in five areas, including agriculture, manufacturing, fisheries, mining and forestry, to enhance transparency. In terms of the temporary movement of natural persons, the parties to the RCEP have committed to facilitate visa access for investors, intra-company transferees, contract service providers, accompanying spouses and family members, and other types of businesspersons from countries in the region who are eligible to enter and obtain residence rights in each country. The RCEP temporary movement of the natural person chapter will facilitate intra-regional commercial labour exchanges and further facilitate the “going out” of national enterprises and the “coming in” of foreign labour [35].

### 2.4. RCEP and COVID-19

The COVID-19 global pandemic caused severe shocks to international trade and global industry chains [36]. In January 2022, China’s Vice Minister of Commerce said that the entry into force of the RCEP would greatly boost confidence in the economic recovery in the context of the epidemic, would effectively hedge the negative impact of COVID-19 on the economy, boost trade and investment confidence, and give a new impetus to the economic growth of member countries. One of the reasons for the Chinese government’s proactive approach to signing the RCEP is the serious impact of COVID-19 on China’s import and export trade, manufacturing chains and service industries [37]. The RCEP is expected to create more jobs in China to alleviate the current huge employment pressure in China [38]. Having considered COVID-19 and the trade war between China and the US, the RCEP could bring huge benefits to China, increasing its trade volume and national income [39]. In China’s Inner Mongolia Autonomous Region, while COVID-19 has had a negative impact on employment well, the signing of the RCEP will offset the negative impact and reap greater benefits [40].

### 2.5. Exogenous Shocks and Employment

The RCEP is a free trade agreement that emerged out of the era of long COVID-19 to counter the shock of COVID-19. Since the outbreak of COVID-19 in late 2019, the dramatic shock has led to a decline in employment levels. This is because the government had to take measures in response to COVID-19, such as total civic silence [41], the closure of workplaces [42], the cessation of public transport [43]. Added to this was the psychological panic caused by COVID-19 [44], all of which led to a chilling winter for employment. A social accounting matrix (SAM) is considered to be an effective way of describing the impact of exogenous shocks on employment [45,46,47]. The effects of exogenous shocks are transmitted through production or consumption linkages. Production linkages depend partly on the extent and intensity of inputs from other sectors that are used to produce the output of the sector initially affected by the shock (i.e., backward linkages). The extent to which the output of the affected sector is used as an input to the production of the upstream industry is also a determinant of production linkages (forward linkages) [48]. In examining how sectoral growth affects inequality, the SAM’s wage accounts were disaggregated into three education levels and ten employment sectors, showing that only growth in agriculture reduced inequality, while growth in the heavy manufacturing and service sectors increased inequality [49]. The SAM is used to examine the impact of public job creation in social care provisioning on employment creation, and the provision of social care also contributes to gender equality [50]. The regional social accounting matrix allows for an economic analysis of the impact of exogenous shocks to entrepreneurial activity on the economy of a region, the sustainability of the economy, economic growth and the contribution to reducing unemployment in the short term [51]. Based on post-Keynesian theory, the social accounting matrix is used to examine changes in demand and household income, and the conclusions suggest that the state becoming the employer of last resort will help reduce unemployment in the mainstream approach, with female income multipliers playing a role in both supply and demand [52]. The social accounting matrix (SAM) has empirical applications in several areas including tourism [53,54,55], energy [53,56,57], environment [58,59] and employment [60,61]. In the social accounting matrix (SAM) for India, it is noted that the labour demand multipliers are higher in agriculture and services. Therefore, these two sectors play a key role in employment generation in the Indian economy [62]. In the SAM study in Ecuador, modelling a 10 percent injection of international tourism demand led to a significant multiplier effect of tourism on the economy and the potential for significant benefits to labour-intensive sectors [63].

## 3. Research Methodology

### 3.1. Social Accounting Matrix

The RCEP and its related government policies have a huge impact on the existing economic system. Most policies directly or indirectly affect the economic system and the labour market, as anyone can benefit or lose from them. It is, therefore, natural for China, which occupies an important position in the global industrial chain and supply chain, that one affected sector will have an impact on other sectors. For example, when RCEP policies affect the grain industry, they may have an impact on the upstream fertiliser industry through the supply chain. How to model the impact of exogenous economic shocks on the economy? Economists consider the social accounting matrix (SAM) model to be an effective tool for dynamically predicting the impact of exogenous shocks on the overall economic system [64,65,66]. Given China’s current strict COVID-19 embargo, capital flows are clearly restricted. It is difficult for the RCEP to work through the endogenous market mechanism of prices. In addition, SAM can find links between one sector and another where RCEP can have a complex impact on the economic system. Results and policy changes are transparent.

To accurately estimate the amount of job creation or destruction that the RCEP may have on multiple sectors in China, and to ensure that policies and outcomes are directly linked, SAM was used in this analysis. The SAM is a square database of transaction matrices, i.e., there are an equal number of rows and columns containing information representing the economy [47]. The square transaction matrix combines an input-output table describing production with national income and production accounts. SAM provides a comprehensive picture of the economic cycle in an economic system in which production creates income, income generates demand, and demand leads to production [67]. Each cell shows the payments from its column to its row. Accounts can be divided into endogenous accounts (e.g., activity, commodities, and factors) and exogenous accounts (e.g., government and fixed capital formation). SAM does not require complex programming knowledge, and the arithmetic allows a transparent indication of how the results are linked to policy changes. As a database, SAM can be used for different types of research, using different analysis methods. One such method is backward linkages analysis, developed by Nobel Prize-winning economist Wassily Leontief [68,69]. Backward linkages reflect the additional intermediate demand generated upstream by expanding production in one sector due to the interdependence between economic sectors and actors. The impact can be due to an exogenous monetary transfer into a sector (i.e., injection) or outflow from a sector (i.e., leakage) on the upstream sector of the economy [70,71]. In this paper, this refers to the entry into force of the RCEP. The indirect effect of an exogenous shock is through production or consumption. The backward linkages through production linkages are the main ones considered in this paper.

The specific operation is as follows: The square transaction matrix is defined as T. The elements in the matrix are defined as $T_{ij}$, where i and j are the individual sectors. Let $T_{en}$ represent the endogenous account and $T_{ex}$ the exogenous account. I represent the identity matrix, with all the diagonal elements of the matrix equal to 1. Dividing each element $T_{ij}$ by the corresponding column sum of the endogenous account ($Y_{en}$), we obtain the technical coefficient of the endogenous account matrix (A). Then, by definition:

$$Y_{en}=T_{en}+T_{ex}=A•Y_{en}+T_{ex}$$

The value of $Y_{en}$ is calculated as follows:

$$Y_{en}={\left(I-A\right)}^{-1}•T_{ex}$$

It may be noted that the matrix ${\left(I-A\right)}^{-1}$ is the famous Leontief inverse matrix. Another important matrix we need is the employment matrix Z. Z is obtained by dividing the number of people employed in each sector by the value of their output and placing these calculated values on the diagonal of the matrix. The final employment effect is matrix E:

$$E=Z•{\left(I-A\right)}^{-1}$$

### 3.2. Model Scenario Design

According to our summary of the RCEP, the entry into force of the RCEP will have a comprehensive impact on the Chinese economy in terms of trade in goods, trade in services, and investment. In comparison, the Comprehensive and Progressive Trans-Pacific Partnership (CPTPP) is a more comprehensive free trade agreement, as it contains both trade-related issues and provisions for deeper integration of member countries, such as state-owned enterprises and designated monopolies, labour, regulatory consistency, transparency, and anti-corruption [72,73,74]. In contrast, the text of the RCEP agreement is more focused on trade-related issues such as trade in goods, rules of origin, and trade in services, while the annexes include a tariff schedule of commitments and a schedule of specific commitments for services, making it a more trade-oriented agreement. Therefore, without loss of generality, the simulated RCEP positively impacts scenarios on China’s labour market in the 149 sectors of the SAM, mainly focused on manufacturing, including mining, processed agricultural and sideline products such as vegetables and fruits, textiles, paper, plastic products, ceramic products, base metals, construction, real estate, wholesale, retail, warehousing and postal services, leasing, and business services (29 sectors in total). China’s Ministry of Commerce estimates that the RCEP will increase China’s real GDP by 0.35 percent more than the baseline, exports by 7.59 percent and imports by 10.55 percent. In addition, simulations by academics at the China Development Research Institute studying the economic impact of the RCEP on sectors in China are around 10% [21]. Therefore, the positive injection rate for exogenous shocks was determined to be 10 percent.

In contrast, because crops are a relatively weak sector in China and a labour-intensive industry, we consider the agricultural products sector as a negative injection [75,76]. For other sectors with negative injection, according to our summary of the RCEP and Zhou Ling Ling’s latest theory of the RCEP’s extensive fan decomposition for different sectors in China, we think that the export market effect of steel, non-ferrous metals and alloys, iron and ferroalloys, metalworking machinery, automotive parts and accessories, electrical equipment and other manufactured products is low. At the same time, the domestic market effects on these sectors are shown to be detrimental [21]. Even if the RCEP is considered a possible response to the shock of COVID-19, the negative impact of COVID-19 on the sector cannot be ignored [77]. Considering the market squeeze caused by the RCEP in some sectors and the shock of COVID-19 in some sectors [78,79,80], a total of 12 sectors are considered as the injection of possible negative effects (X) of the RCEP, with a negative injection of −10 percent.

The final employment effect (Emp) is calculated as follows:

Emp=E•X

### 3.3. Data

The 2017 SAM was compiled from input-output tables published by the National Bureau of Statistics of China. There are 149 sectors in the SAM, including five agricultural sectors such as crops, forestry, livestock, fisheries, and agricultural services, 86 manufacturing sectors, 13 other industrial sectors, and 45 service sectors. Households are separated into two types: rural and urban. Using labour force employment data by industry and labour force data by education from the 2017 China Labour Statistics Yearbook and the 2017 China Population Statistics Yearbook, the labour force was divided into unskilled and skilled workers, supplemented with the latest data from 2020 [81,82]. Based on the actual experience in China, the labour force with junior secondary education or equivalent is considered unskilled workers, while those with senior secondary education or above are classified as skilled workers [83,84,85,86,87]. We calculated the number of employed persons using total wages from the input-output tables and wage rates by industry from the China Statistical Yearbook to measure labour force employment. Adjustment was then made to the number of employed persons in the labour force in each industry using statistical labour force employment in three sectors: agriculture, industry, and services.

## 4. Results

### 4.1. Overall Potential of RCEP for Creation of Jobs

The RCEP is expected to create a significant amount of sectoral employment in China, and even if job creation is much greater than job destruction, the negative impact is still not negligible. In some sectors, such as construction installation, civil engineering construction and building construction, the RCEP is expected to generate a rate of growth in job creation of over 9%. Overall, the RCEP has had a dramatic impact on the Chinese labour market, and it may lead to the estimated potential creation of over 17 million jobs, for China, particularly in some manufacturing industries. It is the consensus of many scholars that the RCEP is likely to bring about a significant increase in employment in China. This is because a country’s economic growth is mainly driven by the “troika” of investment, consumption and exports. It so happens that the RCEP, currently the world’s largest free trade agreement, covers a large area of China in these areas, especially trade. This has injected new economic dynamism into China, a major manufacturing country, especially at the lower and middle end of the manufacturing spectrum. Table 1 details the rate and number of estimated potential job creation or destruction by the RCEP as an exogenous shock variable in 149 sectors in China, in descending order of job growth rate.

### 4.2. Potential of Job Creation According to Workforce Skills

The RCEP is not a perfect free agreement at the moment, and the amount of job creation expected to result is more concentrated in unskilled labour jobs [12]. This also has a lot to do with the fact that China occupies the middle and lower end of the world manufacturing chain [88]. Benefiting from job creation at the lower end of the sector and completing the upgrade from unskilled to skilled labour in a timely manner is key to quality employment in China. The potential job creation of the RCEP in China is quantitatively significant across the skilled labour force. The number of unskilled labour jobs potentially created by the RCEP is about 10.44 million, much larger than the 6.77 million skilled labour jobs. Why is there a 3.67 million gap? The main reason for this is that China’s current industrial structure mainly focuses on products at the middle and lower end of the market. In other words, many jobs are currently in the unskilled labour category. This is also related to the RCEP agreement on China which is mainly about trade in the low-end product sector, which directly leads to the creation of more jobs for unskilled labour than for skilled labour.

### 4.3. The Potential of Job Creation According to the Sectors

It is clear to see that the RCEP has had the most significant impact on job creation in China’s construction installation, civil engineering construction, building construction, gypsum and cement products, cement, lime and gypsum, building materials, building decoration services, non-metallic mining, refractory products and ceramics sectors, with potential job growth rates of over 6 percent. This is related to the volume and growth rate of China’s industry and engineering in the RCEP region, both domestically and abroad. The Chinese government has been committed to infrastructure and engineering labour, especially in the last two decades. From a volume perspective, the potential number of jobs created in sectors such as building construction, civil engineering construction, crops, financial services, retail, professional technical services, wholesale, business services, and forestry exceeded 500,000. There may be a partial sector-to-growth mismatch because of the large labour force (over 100 million) in some specific sectors in China (e.g., agriculture). In general, this is in line with the fact that China’s infrastructure-related sectors are characterised by a large number of employees, large volumes and fast growth rates. For example, in 2020, China’s contracted infrastructure projects in the RCEP region had a completed turnover of about USD 39.19 billion, with an average annual growth rate of 9.1 percent, accounting for 25 percent of China’s total foreign contracting. Among them, ASEAN countries and Australia dominate, at USD 34 billion and USD 3.9 billion, respectively. Contracting work to South Korea was worth USD 730 million, to Japan USD 400 million, and New Zealand USD 100 million. This should also prove that the entry into force of the RCEP will have a more profound impact on China’s infrastructure-related industries.

### 4.4. Negative Effects of RCEP

The damaging effects of COVID-19 on employment remain, and because the RCEP has not had a positive impact on the number of jobs in all sectors, this has allowed the RCEP to increase the restrictiveness of employment in some sectors as well. In some sectors where the domestic market has been squeezed as a result of the RCEP, the RCEP is not a solution to the COVID-19 shock. The metalworking machinery sector was the most severely negatively hit in our simulations. Estimated potential job growth was −5.72 percent, with the potential number of unskilled jobs decreasing by 33,639 and the potential number of skilled jobs decreasing by 22,542. Several sectors that are largely immune to the impact of the RCEP are public administration and organisation, other general equipment, TV and radar equipment, complete vehicles, social work, and hygiene. This is because most of these commitments are related to China’s political and security interests, such as core technologies, scientific research, information technology, social work, biological resources protection, ethnic minorities, special groups, and NGOs. At the same time, state-owned enterprises, emerging companies and sectors are also retained, aiming at a higher degree of autonomy and relatively flexible policy space for China’s future economic development.

## 5. Conclusions

When the world’s largest free trade area agreement meets China, a country rich in labour resources, there is no doubt that it will create violent sparks. The impact of the RCEP on the output of different sectors varies greatly, mainly because of the different degrees of labour intensity in China, the different values of output of different sectors, and the different degrees of participation of different sectors in global value chains. On the whole, China needs to cooperate with other members of the RCEP to exploit its strengths, but also needs to compete to induce the transformation and upgrading of some Chinese sectors in the new free trade agreement (FTA) landscape.

Further exploiting the advantages of labour resource endowment. In this study, the positive impact of RCEP on China’s labour market is mainly concentrated in the construction, real estate and related sectors. The seven sectors where the RCEP is expected to generate the highest growth rates of employment in China are, building decoration services, building materials, cement, lime and gypsum, gypsum and cement products, building construction, civil engineering construction, and construction installation; all of these sectors are expected to grow at over 8%, with a combined increase of around six million. At the same time, this impact will also extend to upstream sectors through backward linkages, such as gypsum, cement products and similar products, building materials such as bricks and stones, building decoration, decoration and other building services. For instance, the Chinese government should take the first year of the RCEP as an opportunity to expand further the number of jobs in related sectors that will be positively affected by the RCEP. Firstly, Chinese sectors such as high-speed rail, steel, building materials and construction should play a leading role in driving the supporting sectors to participate in the RCEP regional cooperation on production capacity and equipment manufacturing, to complete infrastructure projects with high quality and form a comprehensive competitive advantage in the international market. This will stimulate construction and other advantageous sectors to export, while helping RCEP member countries lagging in infrastructure to modernise their infrastructure construction. Secondly, most of the sectors positively affected by the RCEP are labour-intensive industries. The RCEP is expected to create over 10 million unskilled labour jobs in China, particularly in sectors such as crop, forestry, livestock, fishery and mining, which require a lot of labour rather than skills and equipment. It is necessary to speed up the construction of a labour interest protection and risk warning and prevention system. It is also necessary to step up efforts to protect the labour force in important trade routes and major investment projects and effectively prevent and resolve various risks in “going out” investment and international operations to safeguard the personal and asset safety of the labour force. Thirdly, sectors positively affected by the RCEP should, in turn, actively participate in the RCEP rules to facilitate future adaptation to the Comprehensive and Progressive Trans-Pacific Partnership (CPTPP) and other similar rules. By doing so, they will serve as a good model for other sectors to take the lead and will then be able to add to the “new pattern of multilateral trade and investment” opening in China.

Promote industrial upgrading and create more skilled labour jobs. China’s industrial structure is currently in a transition period. Since China’s reform and opening-up in 1978, its economy has lacked competitiveness in the international arena, so it relied on cheap labour to formulate an export-oriented economic policy. Today, China has become the largest industrial country in the world. From the perspective of quantity, China handed over a satisfactory answer sheet. However, in terms of quality, China lags far behind developed countries. How to solve the current problems? The RCEP may be the answer. First, the RCEP can promote China’s industrial upgrading to improve the quality of labour force creation. The accumulation rules of the RCEP’s region of origin are beneficial for multinational companies to rely on the resource endowments and market advantages of each member in the RCEP region to adjust the supply chain layout of the industrial chain and realize efficient allocation of factor resources in the region more flexibly. This will also promote the trade and investment of intermediate products in the region and promote the formation of a closer, stable, and competitive regional industrial chain division and cooperation system. The RCEP also helps to promote the optimal allocation of production factors in the whole region and serves the upstream and downstream industry enterprises in the value chain of final local consumption, which is expected to receive further investment attention. In particular, it will further promote the investment layout of infrastructure, electronic information, petrochemical, textile and garment, automobile and other industries in the RCEP and form a relatively complete industrial chain division pattern covering upstream and downstream, which will also create more jobs for skilled labour. Moreover, China can promote labour-intensive industries or production links to further transfer to other member countries with lower land and labour costs according to RCEP rules of regional accumulation of origin, thereby accelerating the reconstruction of the RCEP regional industrial chain supply chain. However, this may bring some competitive pressure to underdeveloped areas such as central and western China.

Nirvana for sectors negatively affected by RCEP in the long COVID-19 era. Because of the impact of COVID-19 and China’s stringent response policies, labour is not naturally mobile by default, leaving some sectors deeply embedded in the RCEP regional chain negatively affected. For example, the metalworking machinery sector in China has been hit by the synergy of the RCEP agreement and COVID-19, and has seen a decline in jobs. Then again, some agricultural and livestock products and their processing sectors will also be hit by COVID-19 and the RCEP, and jobs in these sectors will likely decline. As workers involved in agricultural production probably know, the most critical factor in the agricultural sector is price. There are still many small workshops in China in the production of agricultural, pastoral, and livestock products, while more large-scale production is taking place abroad. For example, Australia and New Zealand in the RCEP have the advantage of resources for agricultural products, and their agricultural products are relatively cheaper and of better quality. When tariffs fall, consumers will be more inclined to buy from these regions. As such, some of China’s producers of agricultural and livestock products will be affected or may even have to cut production, and jobs will fall. Faced with such a dilemma, the Chinese government must first and foremost avoid pricing out the land and labour factors from the industrialisation model. This also fundamentally protects employment in vulnerable sectors in China. Secondly, policies should be formulated to liberalise the market in these sectors to a certain extent. It is essential to ensure that products with advantages in the RCEP region can enter China smoothly, such as crops from Australia and New Zealand and fruits from ASEAN. It is also vital to ensure that Chinese labour is transferred to a high-tech industrialised model. Finally, cooperation between countries should be strengthened in the post-epidemic era; the signing of the RCEP has had a catalytic effect in enhancing the post-epidemic economic recovery and long-term prosperity of countries. The reduction in tariffs will allow for smoother international trade between member countries, leading to a gradual increase in total international trade and strengthening economic ties between countries, providing a sustained impetus to employment in sectors involved in foreign trade in China.

## 6. Limitations and Future Research

This paper has yet to be verified in terms of the results data, as the RCEP came into force in January 2022 and the number of jobs expected to be created may not be available until 2024. In addition, the social accounting matrix for China in 2021 may likewise need to be reviewed in 2024 due to the data lag in the input-output tables. Future research will focus more on the updating of data and the application of the latest research methods. In addition, the SAM approach can be used to study the impact of exogenous shocks on the economy as a whole, for example how much it affects consumption and the impact of open and closed loops.

## Figures and Tables

**Table 1:** Estimated potential number of 149 sector jobs in China hit by RCEP.

Sector	Growth Rate	Unskilled Labour	Skilled Labour	Sector	Growth Rate	Unskilled Labour	Skilled Labour
Metalworking machinery	−5.72	−33,639	−22,542	Capital markets services	1.40	11,470	89,121
Research and experimental development	0.00	0	0	Office machinery	1.42	2457	1647
Sports	0.02	101	218	Alcohol and wine	1.44	9236	6189
Software services	0.03	342	2353	Mining-specific equipment	1.44	17,740	11,888
Public administration and organisation	0.04	2647	13,886	Air passenger transport	1.44	7490	5611
Other general equipment	0.07	1384	928	Catering	1.46	68,569	51,427
TV and radar equipment	0.07	304	204	Air cargo transportation	1.48	7812	5853
Complete vehicles	0.09	1232	826	Information technology devices	1.51	3513	24,184
Social work	0.12	160	927	Accommodation	1.53	32,759	24,569
Hygiene	0.21	6448	37,228	Technology application services	1.54	5437	32,775
Arts and culture	0.27	984	2133	Recorded reproductions	1.66	19,866	13,313
Leather products	0.30	2468	1654	Other electronic equipment	1.66	4901	3284
Shoes	0.31	2881	1931	Tobacco	1.72	5544	3715
Education	0.32	11,821	85,320	Rubber products	1.74	14,098	9447
Ships and related installations	0.34	2505	1679	Refined Tea	1.74	4514	3025
Knitting or crochet products	0.35	845	566	Instrumentation	1.87	15,855	10,625
Social security	0.39	798	4189	Other manufacturing products	1.88	4535	3039
Communication equipment	0.40	11,623	7789	Water production	1.90	4742	11,000
Specialised agricultural machinery	0.41	1154	773	Paper products	1.92	19,780	13,255
Railway transport equipment	0.42	1495	1002	Telecommunications	1.95	8675	59,724
Public facilities management	0.44	4895	4090	Business Services	2.01	181,133	391,088
Dairy	0.46	1809	1212	Multimodal transport	2.08	56,913	42,641
Medical products	0.47	12,929	8664	Rail passenger transport	2.08	24,066	18,031
Motors	0.47	2681	1797	Non-ferrous metals and alloys	2.10	30,524	20,455
Boilers and original equipment	0.47	2250	1508	Retail	2.17	394,791	374,768
Household appliances	0.50	3535	2369	Transmission and distribution equipment	2.18	30,339	20,331
Non-metal processing special equipment	0.55	3120	2091	Carriage of goods by water	2.27	17,707	13,266
Woollen Textiles	0.56	951	637	Postal services	2.29	71,532	53,594
Fabricated textiles	0.57	2990	2004	Non-ferrous metal mining	2.36	13,474	14,195
Other electrical machinery	0.58	2566	1719	Non-ferrous rolling	2.36	37,942	25,426
Resident services	0.59	34,706	20,026	Plastics	2.39	53,548	35,884
Furniture	0.61	7600	5093	Road transport of goods	2.45	155,760	116,701
Crafts	0.61	3114	2087	Real Estate	2.47	114,491	230,285
Cotton	0.62	14,885	9975	Stevedoring and storage	2.48	20,985	15,723
Press and publications	0.62	1628	3529	Chemical raw materials	2.54	26,626	17,843
Material handling equipment	0.63	2863	1919	Other services	2.56	132,558	76,489
Household chemicals products	0.65	2164	1450	Internet and related services	2.60	8651	59,558
Electronic components	0.67	19,046	12,763	Rail freight transport	2.84	23,086	17,297
Other foods	0.68	11,616	7784	Equipment repair services	2.86	6229	4174
Fishery	0.69	141,037	10,444	Oil and gas extraction	2.94	32,042	33,756
Fishery products	0.70	3953	2649	Pipeline transport	3.06	2220	1663
Meat processing	0.72	9577	6418	Financial services	3.06	90,719	704,873
Feed processing	0.74	4782	3205	Mining activities	3.12	16,991	17,900
Satellite transmission services	0.74	290	1997	Wholesale	3.16	317,644	301,534
Condiments	0.74	1583	1061	Refined petroleum products	3.20	16,712	11,199
Livestock	0.75	308,723	22,861	Synthetic materials	3.29	20,642	13,833
Hemp and silk textiles	0.77	1332	892	Electricity and heat production	3.29	87,746	203,564
Other transport equipment	0.82	4501	3016	Metalwork	3.31	160,566	107,601
Film and video production	0.82	4908	10,641	Iron and ferroalloys	3.39	14,842	9946
Agri-food processing	0.84	8557	5735	Glassware	3.40	29,138	19,526
Other specialised equipment	0.84	14,766	9895	Specialised chemical products	3.53	41,602	27,879
Batteries	0.85	4081	2735	Recycled and processed waste resources	4.18	44,234	29,643
Instant food	0.85	2876	1928	Pigment products	4.20	16,286	10,913
Sugar and sugar products	0.85	964	646	Woodworking products	4.37	58,206	39,006
Audio-visual equipment	0.85	4117	2759	Coal mining and washing	4.38	159,880	168,435
Computers	0.87	12,583	8432	Wire and cable equipment	4.40	47,749	31,998
Textiles clothing	0.87	23,227	15,565	Forestry	4.66	479,868	35,535
Chemical fibre	0.89	4132	2769	Non-metallic mineral products	4.86	22,023	14,758
Grain milling	0.90	7989	5354	Coal Products	4.93	25,225	16,904
Waterborne passenger transport	0.91	324	243	Steel	5.14	20,388	13,662
Entertainment supplies	0.96	12,185	8165	Leasing	5.51	11,614	25,077
Crops	1.00	1,231,799	91,216	Ferrous metal mining	5.51	38,557	40,620
Pesticides	1.02	1790	1199	Steel Rolling	6.06	181,122	121,376
Agricultural services	1.03	35,304	2614	Professional technical services	6.66	103,970	626,700
Fertilizer	1.04	7317	4903	Ceramics	6.78	60,683	40,665
Drinks	1.04	8997	6029	Refractory products	6.85	35,148	23,554
Automotive parts and accessories	1.08	26,480	17,745	Non-metallic mining	7.34	79,935	84,212
Gas production	1.09	2994	6946	Building decoration services	8.32	296,109	88,814
Insurance	1.10	6189	48,092	Building materials	9.44	117,439	78,700
Water resources management	1.12	3668	3065	Cement, lime and gypsum	9.60	100,724	67,498
Ecological protection	1.13	1654	1382	Gypsum and cement products	9.63	146,638	98,267
Pumps, valves, compressors	1.16	7888	5286	Building construction	9.96	2,442,416	732,574
Vegetable oil processing	1.19	5866	3931	Civil engineering construction	9.96	1,199,305	359,717
Urban public transport	1.29	36,622	27,439	Construction installation	9.97	141,700	42,501
Entertainment	1.38	7916	17,163				

## Data Availability

https://data.stats.gov.cn/ifnormal.htm?u=/files/html/quickSearch/trcc/trcc01.html&h=740 (accessed on 24 October 2022).
